# Selection and early clinical evaluation of the brain‐penetrant 11β‐hydroxysteroid dehydrogenase type 1 (11β‐HSD1) inhibitor UE2343 (Xanamem™)

**DOI:** 10.1111/bph.13699

**Published:** 2017-01-25

**Authors:** Scott P Webster, Andrew McBride, Margaret Binnie, Karen Sooy, Jonathan R Seckl, Ruth Andrew, T David Pallin, Hazel J Hunt, Trevor R Perrior, Vincent S Ruffles, J William Ketelbey, Alan Boyd, Brian R Walker

**Affiliations:** ^1^ Centre for Cardiovascular ScienceUniversity of Edinburgh, Queen's Medical Research InstituteEdinburghUK; ^2^ Charles River LaboratoriesHarlowUK; ^3^ Corcept TherapeuticsMenlo ParkCaliforniaUSA; ^4^ Domainex LimitedCambridgeUK; ^5^ Actinogen Medical LtdSydneyNew South WalesAustralia; ^6^ Alan Boyd Consultants LtdCreweUK

## Abstract

**Background and Purpose:**

Reducing glucocorticoid exposure in the brain via intracellular inhibition of the cortisol‐regenerating enzyme 11β‐hydroxysteroid dehydrogenase type 1 (11β‐HSD1) has emerged as a therapeutic strategy to treat cognitive impairment in early Alzheimer's disease (AD). We sought to discover novel, brain‐penetrant 11β‐HSD1 inhibitors as potential medicines for the treatment of AD.

**Experimental Approach:**

Medicinal chemistry optimization of a series of amido‐thiophene analogues was performed to identify potent and selective 11β‐HSD1 inhibitors with optimized oral pharmacokinetics able to access the brain. Single and multiple ascending dose studies were conducted in healthy human subjects to determine the safety, pharmacokinetic and pharmacodynamic characteristics of the candidate compound.

**Results:**

UE2343 was identified as a potent, orally bioavailable, brain‐penetrant 11β‐HSD1 inhibitor and selected for clinical studies. No major safety issues occurred in human subjects. Plasma adrenocorticotropic hormone was elevated (a marker of systemic enzyme inhibition) at doses of 10 mg and above, but plasma cortisol levels were unchanged. Following multiple doses of UE2343, plasma levels were approximately dose proportional and the terminal *t*_1/2_ ranged from 10 to 14 h. The urinary tetrahydrocortisols/tetrahydrocortisone ratio was reduced at doses of 10 mg and above, indicating maximal 11β‐HSD1 inhibition in the liver. Concentrations of UE2343 in the CSF were 33% of free plasma levels, and the peak concentration in CSF was ninefold greater than the UE2343 IC_50_.

**Conclusions and Implications:**

UE2343 is safe, well tolerated and reaches the brain at concentrations predicted to inhibit 11β‐HSD1. UE2343 is therefore a suitable candidate to test the hypothesis that 11β‐HSD1 inhibition in brain improves memory in patients with AD.

**Abbreviations**
ACTH: adrenocorticotropic hormone
AD: Alzheimer's disease
Ae: amount excreted
Aβ: amyloid β
b.i.d: twice daily
BMI: body mass index
CL_R_: renal clearance
C_max_: maximum plasma concentration
C_min_: minimum plasma concentration
CV: coefficient of variation
CYP450: cytochrome P450
DHEA‐s: dehydroepiandrosterone sulphate
HPA: hypothalamic–pituitary‐adrenal
HSD: hydroxysteroid dehydrogenase
MPO: multiparameter optimization
q.d: once daily
TEAEs: treatment‐emergent adverse events
TH: tetrahydro
THE: tetrahydrocortisone
THF: tetrahydrocortisol
T_max_: time to maximum plasma concentration

## Tables of Links

**Table:** 

**TARGETS**	
**Voltage‐gated ion channels** ^*a*^	**Enzymes** ^*c*^
hERG (K_v_11.1) channels	3α‐reductase (AKR1C3)
**Nuclear hormone receptors** ^*b*^	5α‐reductase
Glucocorticoid receptor (GR)	11β‐HSD1
Mineralocorticoid receptor (MR)	CYP1A2
	CYP2C9
	CYP2C19
	CYP2D6
	CYP3A4

**LIGANDS**
4‐androstenedione
ACTH
Amyloid β
Cortisol
Cortisone
DHEA‐s
Testosterone

These Tables list key protein targets and ligands in this article which are hyperlinked to corresponding entries in http://www.guidetopharmacology.org, the common portal for data from the IUPHAR/BPS Guide to PHARMACOLOGY (Southan *et al*., 2016), and are permanently archived in the Concise Guide to PHARMACOLOGY 2015/16 (^*a,b,c*^Alexander *et al*., 2015a,b,c).

## Introduction

Chronically elevated levels of the glucocorticoid stress hormone cortisol are associated with a variety of conditions including Cushing's syndrome, which is characterized by the metabolic complications of central obesity, dyslipidaemia, hypertension and glucose dysregulation (Anagnostis *et al.,* 2009). In Cushing's syndrome, neuropsychiatric problems also occur, including cognitive impairment, which is related to excess cortisol exposure in key areas of the brain responsible for memory (Starkman *et al.,* 1999). Increased glucocorticoid exposure in the brain is linked to age‐related cognitive decline where cortisol levels align with reductions in hippocampal volumes and consequent memory impairment (Lupien *et al.,* 1998; MacLullich *et al.,* 2005; Lupien *et al.,* 2009). More subtle alterations in glucocorticoid levels, exemplified by higher morning cortisol levels, have also been shown to be associated with impaired cognitive performance (Reynolds *et al.,* 2010).

In Alzheimer's disease (AD), elevated plasma cortisol levels are associated with accelerated disease progression, while higher cortisol levels in CSF are associated with more rapid clinical worsening and cognitive decline in mild cognitive impairment of the AD type (Cernansky *et al.,* 2006; Kornhuber and Jessen, 2015; Popp *et al.,* 2015). Individuals with the *APOE‐*ε4 allele, a key risk factor for AD, have higher CSF cortisol (Peskind *et al.,* 2001). Studies in rodents have provided further evidence that excess glucocorticoids link to disease progression with synthetic glucocorticoid treatment leading to increased amyloid β (Aβ) formation, reduced Aβ degradation and increased τ expression in the brain (Green *et al.,* 2006). Reducing glucocorticoid action may therefore be beneficial in the treatment of AD.

Directly manipulating the hypothalamic–pituitary‐adrenal (HPA) axis in AD is not attractive, since a reduction in circulating cortisol risks impairing the stress response. However, tissue‐specific modulation of intracellular cortisol levels without concomitant reductions in plasma cortisol can be achieved by inhibiting the cortisol‐generating enzyme 11β‐hydroxysteroid dehydrogenase type 1 (11β‐HSD1) in relevant brain areas including the hippocampus. Pharmacological inhibition of 11β‐HSD1 leads to reduction in Aβ plaques in the brains of Tg2576‐AD mice and to improvements in memory (Sooy *et al.,* 2015). In aged rodents, modulation of 11β‐HSD1 by genetic knockdown or pharmacological inhibition improves memory (Yau *et al.,* 2001; Sooy *et al.,* 2010; Mohler *et al.,* 2011; Yau *et al.,* 2014), while treatment of cognitively impaired healthy elderly men and patients with type 2 diabetes, with the non‐selective 11β‐HSD1 inhibitor carbenoxolone, has been shown to improve memory (Sandeep *et al.,* 2004). Lowering cortisol levels in the brain via inhibition of 11β‐HSD1 has thus emerged as a therapeutic strategy for the treatment of cognitive impairment in early‐stage AD (Reynolds and Webster, 2015).

Here, we report the discovery of the brain‐penetrant 11β‐HSD1 inhibitor UE2343 and the results of Phase 1 clinical studies to establish its safety, pharmacokinetics, distribution to the brain and tissue‐specific pharmacodynamics.

## Methods

### Chemical synthesis

The synthetic methods for the preparation of compounds **1** to **9** are reported in patent applications WO2011033255 and WO2011135276 (Webster *et al.,* 2011a,b).

### 11β‐HSD1 inhibition

Inhibition of human 11β‐HSD1 was determined in HEK293 cells stably transfected with a construct containing the full‐length gene coding for the human 11β‐HSD1 enzyme according to the protocol described by Sooy *et al.,* 2010.

### Microsomal stability

Microsomal stability was determined using pooled human, rat or dog liver microsomes according to the method described in WO2011135276 (Webster *et al.,* 2011a).

### Plasma protein binding

Plasma protein binding was determined using pooled human plasma according to the method described in WO2011135276 (Webster *et al.,* 2011a).

### hERG inhibition

Testing was performed by Essen Instruments, Hertfordshire, UK. Compounds were tested for inhibition of the human ether a go‐go related gene (hERG) K+ channel in a Chinese hamster lung cell line stably expressing the full length hERG channel. Single cell ionic currents were measured in the perforated patch clamp configuration at room temperature using an IonWorks Quattro instrument. Eight‐point concentration–response curves were generated using threefold serial dilutions from the maximum final assay concentration. IC_50_ values were obtained from a four‐parameter logistic fit of the % inhibition data.

### Aqueous solubility

Aqueous solubility was determined by suspending sufficient compound in aqueous buffer (0.1 M potassium phosphate, pH 7.4; 0.15 M potassium chloride) to give a maximum possible final concentration of 1 mg·mL^−1^ of the compound at room temperature. The concentration was quantified by HPLC with reference to a standard calibration curve. The solubility was calculated using the peak areas determined by integration of the peak found at the same retention time as the principal peak in the standard injection.

### Cytochrome P450 inhibition

Testing was performed by Cyprotex Discovery Limited, Cheshire, UK. Six test compound concentrations (0.4, 1, 4, 10, 40 and 100 μM; final DMSO concentration 0.25%, final microsome concentration 0.25 mg·mL^−1^) were either pre‐incubated for 30 min in the absence and presence of NADPH or underwent a 0 min pre‐incubation with human liver microsomes. At the end of the pre‐incubation period, the probe substrate for each cytochrome P450 (CYP450) isoform (30 μM) and NADPH (1 mM) were then added (final DMSO concentration 0.3%), and the samples were incubated for 5 min at 37°C. Probe substrates were as follows: 1A2 – phenacetin, 2C9 – diclofenac, 2C19 – mephenytoin, 2D6 – dextromethorphan, 3A4 – midazolam and testosterone. Samples were subjected to LC–MS/MS analysis to determine metabolite concentrations. A decrease in the formation of the metabolite compared with vehicle control was used to calculate an IC_50_ value (test compound concentration which produces 50% inhibition) for each experimental condition.

### Pharmacokinetics in rat

Pharmacokinetic studies were carried out at Quotient Bioresearch, UK. For both i.v. and p.o. administration, compounds were prepared in 0.9% saline containing 2% DMSO (v^.^v^‐1^) and 38% PEG400 (v^.^v^‐1^). Solutions were passed through 0.22 μm filters prior to administration. Animals were given *ad libitum* access to food and water throughout the study.

For i.v. administration three male Sprague Dawley rats each received 1 mg·kg^−1^ of compound by tail vein injection (dose volume 5 mL·kg^−1^). Serial blood samples were taken from a lateral tail vein 5, 15, 30 min, 1, 2, 4, 6 and 8 h post i.v. administration, and stored in individual EDTA containers. Blood samples were flash frozen in liquid nitrogen and stored at −20°C prior to analysis.

For p.o. administration, five male Sprague Dawley rats were dosed with compound at 5 mg·kg^−1^ (dose volume 10 mL·kg^−1^). Serial blood samples were taken from a lateral tail vein 15, 30 min, 1, 2, 4, 6 and 8 h post p.o. administration, and stored in individual EDTA containers. Blood samples were flash frozen in liquid nitrogen and stored at −20°C prior to analysis.

Blood samples were thawed at ambient temperature and vortex mixed thoroughly. A representative aliquot of each blood sample was diluted 1:1 v^.^v^‐1^ with HPLC grade water prior to protein precipitation with acetonitrile containing diazepam internal standard (blood/water: acetonitrile ratio was 1:4 v^ ^v^‐1^). Following vortex mixing and centrifugation (4°C, 15 min, 17 000 x *g*) a 100 μL aliquot of supernatant was diluted by addition of 50 μL HPLC grade water in a well of a 96‐well midi‐eppendorf plate. The plate was sealed and shaken on a plate shaker to ensure sample homogeneity. Samples were assayed against a series of matrix matched calibration curve standards by LC–MS/MS using a Sciex 3000 mass spectrometer. Pharmacokinetic parameters were derived by non‐compartmental analysis using WinNonLin pharmacokinetic software.

Animals were group‐housed, provided free access to food and water and maintained in accordance with UK Home Office regulations and according to established procedures. Following the final blood sample animals were killed via a Schedule 1 method.

### Pharmacokinetics in dog

Pharmacokinetic studies were carried out at Charles River, UK. For both i.v. and p.o. administration, compounds were prepared in 0.9% saline containing 2% DMSO (v^.^v^‐1^) and 38% PEG400 (v^.^v^‐1^). Solutions were passed through 0.22 μm filters prior to administration. Three Beagle dogs each received a single i.v. injection of compound **4** at a dose level of 1 mg·kg^−1^. Following a washout period of 1 week, each animal received a single p.o. dose of compound **4** at a dose level of 5 mg·kg^−1^.

After a washout period of 6 weeks, the same three dogs each received a single i.v. injection of compound **7** at a dose level of 1 mg·kg^−1^. Following a washout period of 1 week, each animal received a single p.o. dose of compound **7** at a dose level of 5 mg·kg^−1^.

Plasma samples were obtained up to 24 h post dose administration, and the concentration of each test item in plasma was determined using LC–MS/MS. Pharmacokinetic parameters were derived by non‐compartmental analysis using WinNonLin pharmacokinetic software.

During pre‐trial and on‐study periods, animals were group‐housed and maintained according to established procedures as detailed in appropriate standard operating procedures (SOPs) and in accordance with UK Home Office regulations. Except for a period of fasting, pre‐dose to approximately 4 h post‐dose animals were fed a daily allowance of 400 g of standard laboratory diet and mains quality tap water was available *ad libitum* throughout the study. Following completion of the study animals were returned to the colony. The animal work was conducted under UK Home Office Project Licence no. PPL 60/4186.

### Tissue exposure measurements in rats

The circulating plasma levels and tissue distribution of compounds were determined according to the method described in WO2011135276 (Webster *et al.,* 2011a). Animals were group‐housed, had free access to food and water and were maintained in accordance with UK Home Office regulations. The animal work was conducted under UK Home Office Project Licence no. PPL 60/3915.

Compounds were prepared in 0.9% saline containing 2% DMSO (v^.^v^‐1^) and 38% PEG400 (v^.^v^‐1^) and administered to male Sprague Dawley rats by oral gavage such that the final concentration of compound was 10 mg·kg^−1^. Rats (*n* = 3 per time point) were killed by decapitation at 1, 4 and 6 h post dosing, and trunk blood and tissues (liver, adipose and brain) were excised. Blood samples at 0.5 and 2 h post dosing were taken by a tail nick from the 4 and 6 h rats respectively.

Compounds were triple extracted from plasma (prepared from blood by a high speed centrifugation step) and spiked with 1 μg of a standard compound using ethyl acetate. Extracts were dried under nitrogen and re‐suspended in 60% methanol/40% ammonium acetate (5 mM).

A known weight of tissue was homogenized in 3 volumes of Krebs buffer. The compound was triple extracted with ethyl acetate from the supernatant of a low speed spin spiked with 1 mg of a standard compound. Extracts were dried under nitrogen and re‐suspended in 60% methanol/40% ammonium acetate (5 mM).

Samples from plasma and tissue were analysed by TSQ Quantum Discovery Tandem Mass Spectrometer and Surveyor Liquid Chromatogram (Thermo, Hemel‐Hempstead, UK). Then 10 μL of each sample was injected in a mobile phase consisting of 60% methanol/40% ammonium acetate (5 mM) at a flow rate of 0.5 mL·min^−1^. The column used was a BDS hypersil C18, 50 × 2.1 mm with a 5 μm particle size.

Each compound was tuned with a spray voltage of 3000 V and a capillary temperature of 300°C, and values for tube lens, CID and product ions were determined. The peak area for each compound and for the internal standard was determined, and the concentration of compound ^.^ g^‐1^ of tissue or mL^‐1^ of plasma was calculated by comparison of the peak area ratio to a standard curve.

### Compliance with requirements for studies using animals

Studies using animals were carried out in accordance with UK Home Office regulations under appropriate project licences. Pharmacokinetic and tissue exposure measurements were carried out using standard study designs, and the numbers of animals used per study were in line with those required to support regulatory submissions to the UK Medicines and Healthcare products Regulatory Agency. Animal studies are reported in compliance with the ARRIVE guidelines (Kilkenny *et al*., 2010; McGrath & Lilley, 2015).

### Clinical study design and subject characteristics

#### Single ascending dose study

A double‐blind, randomized, placebo‐controlled single ascending dose study was carried out at Simbec Research Ltd, Merthyr Tydfil, UK. Each cohort consisted of six healthy male subjects and two females of non‐child bearing potential. Two male ‘dose leader’ subjects (1 active : 1 placebo) were dosed first in each cohort. The remainder of the cohort (5 active : 1 placebo) were dosed 24 h later. Dose cohorts were 2, 5, 10, 18, 25 and 35 mg. Safety, pharmacokinetic and pharmacodynamic assessments were made at pre‐determined time‐points during each part of the study. Dose escalation was dependent upon satisfactory review of the blinded safety (up to Day 6) and pharmacokinetic (up to 24 h post‐dose) data from the preceding cohort.

#### Multiple ascending dose study

A double‐blind, randomized, placebo‐controlled multiple ascending dose study was carried out at Linear Clinical Research Ltd, Nedlands, Australia. Each cohort consisted of eight healthy male subjects. Each subject received UE2343 or placebo twice daily (b.i.d ) (12 hourly) in the fed state for 9 days (Day 1 to Day 9) and once on the morning of Day 10 (19 doses in total). Dose cohorts were 10, 20 and 35 mg. Dose escalation and selection was dependent upon satisfactory review of the blinded safety (up to Day 17 ± 2) and plasma pharmacokinetics (up to Day 12) data from the preceding cohort. Safety, pharmacokinetic and pharmacodynamic assessments were made at pre‐determined time‐points during each part of the study.

#### CSF study

A non‐randomized, open label, multiple dose phase I study to determine the pharmacokinetic parameters of UE2343 in CSF was carried out at Linear Clinical Research Ltd, Nedlands, Australia. Four healthy male subjects each received 35 mg UE2343 b.i.d (12 hourly) fed (30 min after the start of a standard meal) for 3 days (Day 1 to Day 3) and once on the morning of Day 4 (seven doses in total). Plasma pharmacokinetic samples were taken from each subject on Day 1 and Day 4. A single CSF sample was taken from each subject at 5 h post‐final dose on Day 4, corresponding to the plasma T_max_ (time to maximum plasma concentration).

Approval for each clinical study was obtained following review of protocols by local research ethics committees.

### Analytical methods

Laboratory evaluation of clinical safety parameters was carried out using standard validated methods. Measurement of plasma pharmacodynamic markers [adrenocorticotropic hormone (ACTH), cortisol, 4‐androstenedione, testosterone and dehydroepiandrosterone sulphate (DHEA‐s)] was carried out using commercially available kits. Each method was fully validated prior to analysis. Urinary steroid metabolites were measured according to established methods (Boonen *et al.,* 2013). UE2343 levels in plasma, urine and CSF was determined by LC–MS/MS using fully validated methods.

### Statistical analysis

The data and statistical analysis comply with the recommendations on experimental design and analysis in pharmacology (Curtis *et al.,* 2015). Levels of probability, *P* < 0.05 were deemed to constitute the threshold for statistical significance. Pharmacokinetic parameters, except T_max_ (time of maximum observed drug concentration), are given as mean ± coefficient of variation (CV%). T_max_ is given as median ± range. For each of the pharmacodynamic parameters [ACTH, cortisol, adrenal androgens, tetrahydrocortisols (THFs)/tetrahydrocortisone (THE) ratio], the maximum % change from baseline for each subject was subjected to ANOVA. For selected data, an additional covariate factor for the baseline was also included in the ANOVA/analysis of covariance (ANCOVA) model. Associated *P*‐values <0.05 for each comparison have been presented.

The ACTH levels for each individual given a single dose of UE2343 on Day 1 (0 and 23 h post‐dose) were also subjected to a fixed effects ANOVA, with a fixed effect for dose level. The associated *P*‐values <0.05 between 0 and 23 h post‐dose have been presented.

Unless stated otherwise, data has been plotted as mean ± SEM.

## Results

### Selection of UE2343 for clinical studies

**Figure 1:**
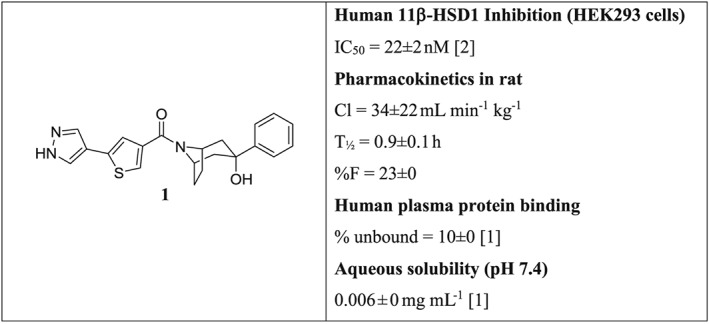
Lead structure and properties. Pharmacokinetics in rats was conducted by i.v. (*n* = 3 rats) and p.o. administration (*n* = 5 rats). The number of individual experiments [*n*] for measurement of *in vitro* human 11β‐HSD1 inhibition, plasma protein binding and aqueous solubility are shown. Data are reported as mean ± SD.

A series of 3,3‐disubstituted‐(8‐aza‐bicyclo[3.2.1]oct‐8‐yl)‐[5‐(1H‐pyrazol‐4‐yl)‐thiophen‐3‐yl]‐methanones were discovered that displayed potent and selective inhibition of human 11β‐HSD1 as exemplified by compound **1** (Figure 1) (Webster *et al.,* 2011a). Final lead optimization focused on improving the physicochemical and pharmacokinetic characteristics of the lead series by examining heterocyclic alternatives to the 3‐phenyl group of compound **1** and by replacement of the 3‐hydroxyl group with fluorine or nitrile (Figure 1). Physicochemical parameters were aligned with those required for brain penetration since potential candidate compounds were required to enter the brain. CNS multiparameter optimization (MPO) calculations were also performed leading to the generation of a subset of compounds with CNS MPO scores ≥5 that were predicted to cross the blood–brain barrier (Table 1) (Wager *et al.,* 2010). All compounds displayed good levels of potency in cellular assays of 11β‐HSD1 (IC_50_ < 50 nM) and selectivity at the isozyme 11β‐HSD2 (IC_50_ > 10 μM, data not shown). Hydroxyl, fluorine and nitrile groups were well tolerated in combination with a variety of heterocycles. Single 3‐exo isomers were obtained for hydroxyl and nitrile analogues, but synthesis of fluorinated analogues (compounds **6** and **7**) led to the preparation of both 3‐exo and 3‐endo isomers (Webster *et al.,* 2011b). The 3‐exo isomer (compound **7**) displayed greater potency towards 11β‐HSD1 than the corresponding 3‐endo isomer (compound **6**) and possessed improved pharmacokinetic properties compared with the 3‐endo isomer, including a twofold greater free fraction in plasma.

**Table 1:** Potency, plasma and brain levels of compounds **1**–**9**

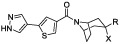							
ID	X	R	IC_50_ (nM) [*n*]	CNS MPO score	Plasma C_max_ (ng·mL^−1^)	Brain C_max_ (ng·mL^−1^)	Brain : plasma ratio
**1**	OH	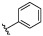	22 ± 2 [2]	5.0	419 ± 28	29 ± 10	0.07
**2**	OH	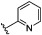	26 ± 8 [3]	5.4	1270 ± 330	31 ± 12	0.02
**3**	OH	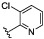	12 ± 0 [1]	5.0	115 ± 48	36 ± 12	0.31
**4**	OH	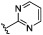	24 ± 15 [31]	5.2	1539 ± 657	173 ± 80	0.11
**5**	OH	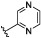	37 ± 3 [2]	5.2	589 ± 161	19 ± 17	0.03
**6**	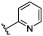	F	22 ± 10 [4]	5.4	399 ± 99	108 ± 54	0.27
**7**	F	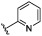	7 ± 4 [5]	5.4	1526 ± 212	374 ± 184	0.24
**8**	CN	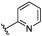	15 ± 0 [1]	5.6	291 ± 74	9 ± 3	0.03
**9**	CN	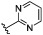	13 ± 3 [3]	5.3	230 ± 33	15 ± 1	0.07

Plasma and brain levels were determined as described in the Methods section using *n* = 3 rats per time point. The number of individual experiments [*n*] for measurement of *in vitro* human 11β‐HSD1 inhibition are shown. Data are reported as mean ± SD.

Plasma and brain partitioning was determined by conducting tissue exposure studies in rats. The greatest brain : plasma ratio was obtained for the pyridine analogues **3**, **6** and **7** (Table 1). However, only the 3‐fluoro‐3‐pyridine analogue **7** displayed sufficient oral bioavailability for further consideration. Moderate CNS exposure was obtained for the 3‐hydroxy‐3‐pyrimidine analogue **4**, which displayed comparable plasma levels to compound **7.** When rat plasma protein binding was taken into account (Table 2) the brain : plasma free ratios were 2.45 for compound **7** and 0.43 for compound **4**, indicating that a greater proportion of compound **7** was also able to enter the CNS.

**Table 2:** Comparative *in vitro* and *in vivo* properties of compounds 4 and 7

Compound	4	7
**Human 11β‐HSD1 IC**_**50**_ **in HEK293 cells (nM) [*n*]**	24 ± 15 [31]	7 ± 4 [5]
**Liver microsomal stability** **(% parent at 30 min) [*n*]**	Human	Human
	99 ± 1 [3]	51 ± 3 [3]
	Dog	Dog
	92 ± 5 [3]	74 ± 1 [3]
	Rat	Rat
	74 ± 1 [3]	45 ± 5 [3]
**Pharmacokinetics in dog**		
**Oral C_max_ (ng·mL^−1^)**	4590 ± 546	3100 ± 908
**Cl (mL·min^−1^·kg^−1^)**	4.68 ± 1.12	8.68 ± 2.03
***t***_**1/2**_ **(h)**	2.79 ± 0.84	3.21 ± 1.61
**Oral AUC_(0➔∞)_ (ng.h·mL^−1^)**	31 730 ± 3746	9208 ± 2005
**% F**	170 ± 40	90 ± 40
**Plasma protein binding** **(% unbound) [*n*]**	Human	Human
	30 ± 2 [2]	11 ± 2 [2]
	Dog	Dog
	44 ± 0 [1]	11 ± 0 [1]
	Rat	Rat
	26 ± 0 [2]	10 ± 1 [2]
**Human hERG IC**_**50**_ **(**μM**) [*n*]**	>30 ± 0 [2]	3.1 ± 0.6 [2]
**Aqueous solubility at pH 7.4 (mg**·**mL** ^**−1**^ **) [*n*]**	0.200 ± 0 [1]	0.006 ± 0 [1]

Pharmacokinetics in dog was conducted by i.v. and p.o. administration (*n* = 3 dogs per route). The number of individual experiments [*n*] for measurement of *in vitro* human 11β‐HSD1 inhibition, liver microsomal stability, plasma protein binding, hERG inhibition and aqueous solubility are shown. Date are reported as mean ± SD.

Further pharmacokinetic comparison of compounds **4** and **7** was performed in dogs, which demonstrated that both compounds exhibited high bioavailability and moderate half‐lives (Table 2). Compound **4** was cleared more slowly than compound **7** in line with its greater *in vitro* stability in dog liver microsomes (Table 2). Strikingly compound **4** gave greater than threefold higher oral exposure than compound **7** and displayed >100% bioavailability. The reasons for a bioavailability value of >100% are unknown and there was no evidence of enterohepatic recirculation.

Although compound **7** demonstrated higher levels in rat brain than compound **4**, profiling of both compounds *in vitro* demonstrated that compound **4** possessed a more attractive profile for further development in humans than compound **7**, including substantially greater stability in human liver microsomes, higher free fraction in human plasma and higher aqueous solubility (Table 2). Crucially, compound **4** displayed only marginal inhibition of the hERG channel up to a concentration of 30 μM, whereas compound **7** was a moderate hERG inhibitor (IC_50_ = 3.1 μM, Table 2). Subsequent *in vitro* profiling of compound **4** revealed a clean off‐target profile in a diversity screen of 29 enzymes and 72 receptors, including the glucocorticoid and mineralocorticoid receptors (data not shown). For compound **4,** no significant CYP450 inhibition was observed at isoforms 1A2, 2D6, 2C9 or 3A4 ((IC_50_ > 50 μM). However, moderate inhibition of isoform 2C19 (IC_50_ = 1.7 μM) was observed. No time‐dependent inhibition was observed at any of the CYP450 enzymes tested. Compound **4** was thus chosen for further development in humans and designated with the code UE2343.

### Subject demographics and safety

A single ascending dose study was conducted in 36 healthy male subjects (35 Caucasians and 1 Afro‐Caribbean) and 12 healthy Caucasian females of non‐child bearing potential. For males, the mean age was 34.1 ± 9.7 years and the mean body mass index (BMI) was 26.0 ± 2.4 kg·m^−2^. For females, the mean age was 49.3 ± 9.1 years and the mean BMI was 25.1 ± 2.1 kg·m^−2^. Each cohort consisted of six males and two females. The incidence of treatment‐emergent adverse events (TEAEs) was considered to be low (number of subjects with ≥1 TEAEs: placebo = 6, UE2343 = 9), and none was associated with any clinically significant changes in vital signs, ECG, biochemistry, haematology or urinalysis data.

A multiple ascending dose study was conducted in 24 healthy male subjects (4 Caucasians and 20 Asians) with a mean age of 26.7 ± 5.4 years and a mean BMI of 23.9 ± 1.9 kg·m^−2^. No serious TEAEs or TEAEs that led to subject withdrawal from the study were reported, and all TEAEs were mild or moderate in intensity. The most common TEAE was headache reported in 7/24 subjects; diarrhoea was reported in 2/24 subjects, and thrombophlebitis was reported in 3/24 subjects.

A study to determine the amount of UE2343 in CSF was conducted in four healthy Asian male subjects with mean age of 29 ± 11 years and mean BMI of 24.6 ± 1.4 kg·m^−2^. All TEAEs were mild to moderate in intensity. Vital signs remained stable during the study, and no out of the range values were recorded for any of the subjects during the study. Increased alanine aminotransferase was reported in a single subject.

### Pharmacokinetics

**Figure 2:**
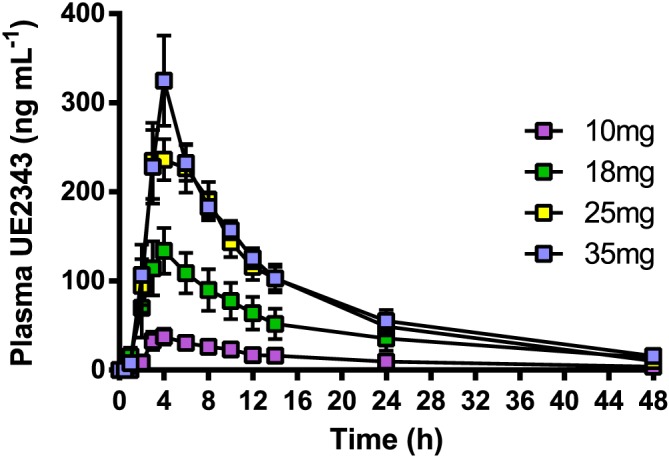
UE2343 concentrations following a single oral dose of UE2343. Six healthy male and two healthy female subjects per dose level.

In the single ascending dose study UE2343 reached a maximum plasma concentration (C_max_) within 3.5–4 h following administration of 10, 18, 25 and 35 mg of UE2343 (Figure 2). Plasma levels of UE2343 at doses of 2 and 5 mg were below the level of quantification, and data are not reported. Plasma UE2343 levels increased with increasing dose and overall exposure, as measured by C_max_ and AUC (AUC_0‐t_ and AUC_0‐∞_), increased in a greater than proportional manner with increasing dose (Table 3). The greatest increase in exposure was observed between 10 and 18 mg UE2343, with an approximate fourfold increase between each of these two doses. UE2343 concentrations appeared to start to plateau between the 25 and 35 mg dose levels. The *t*_1/2_ ranged from 10.5 to 18.7 h and the oral clearance from 7.3 to 8.8 l·h^−1^. Both parameters were dose‐independent following single doses. Urinary excretion of UE2343 accounted for ≤1.8% of the respective doses, with a renal clearance (CL_R_) of <0.13 l·h^−1^. The amount of UE2343 excreted in urine over the 120 h sampling period (amount excreted [Ae]_(0–120h)_), increased with dose in a greater than proportional manner across the 10 mg to the 35 mg dose levels, with the greatest increase observed between the 10 and 18 mg dose levels.

**Table 3:** Pharmacokinetic parameters following a single oral dose of UE2343 in humans

Pharmacokinetic parameter	UE2343			
	10 mg Mean (CV%)	18 mg Mean (CV%)	25 mg Mean (CV%)	35 mg Mean (CV%)
C_max_ (ng·mL^−1^)	40.4 (62%)	136 (46%)	278 (30%)	329 (37%)
T_max_ (h)^a^	3.5 (3–6)	4.0 (3–4)	4.0 (3–6)	4.0 (4–6)
*t*_1/2_ (h)	18.7 (59%)	13.6 (61%)	10.5 (35%)	11.4 (24%)
AUC_0‐t_ (ng^.^h·mL^−1^)	550 (90%)	2291 (87%)	3486 (39%)	4016 (30%)
AUC_0‐∞_ (ng^.^h·mL^−1^)	856 (70%)	2581 (84%)	3825 (35%)	4261 (29%)
Oral clearance (L·h^−1^)	18.5 (75%)	14.5 (90%)	7.32 (38%)	8.78 (28%)
Ae_(0–120h)_ (μg)	52.5 (85%)	300 (66%)	438 (37%)	501 (15%)
Ae_(0–120h)_ (%)	0.53 (85%)	1.67 (66%)	1.75 (37%)	1.43 (15%)
CL_R_ (L·h^−1^)	0.07 (86%)	0.16 (50%)	0.13 (46%)	0.12 (16%)

Single ascending dose studies were conducted in six healthy male and two healthy female subjects per dose level.

a Median ± range

In the multiple ascending dose study, the pharmacokinetic parameters of UE2343 at dose levels of 10, 20 and 35 mg administered every 12 h were determined on Days 1 and 10. Plasma UE2343 levels as measured by C_max_, and AUC over 12 h (AUC_0–12_) were approximately dose proportional on Day 10 (Table 4). However, these parameters increased more rapidly than proportionally to dose level on Day 1. Steady state was achieved by Day 5 and the terminal *t*_1/2_ ranged from 10 to 14 h. The median time to peak concentration following each dose ranged from 4 to 6 h. There was considerable accumulation with 12 hourly dosing, with increase in AUC_0–12_ from first dose to steady state of greater than fourfold at the highest dose level. The Ae in urine was low, with the fraction excreted in the dosing interval approximately 4% at steady state.

**Table 4:** Pharmacokinetic parameters following multiple oral doses of UE2343 in humans

Pharmacokinetic parameter	Day 1			Day 10		
	10 mg UE2343 Mean (CV%)	20 mg UE2343 Mean (CV%)	35 mg UE2343 Mean (CV%)	10 mg UE2343 Mean (CV%)	20 mg UE2343 Mean (CV%)	35 mg UE2343 Mean (CV%)
C_max_ (ng·mL^−1^)	29.1 (61%)	89.2 (71%)	268 (34%)	207 (58%)	365 (29%)	892 (30%)
T_max_ (h)^a^	6.0 (4–8)	4.0 (3–10)	4.0 (3–6)	4.0 (3–6)	3.0 (2–6)	4.0 (3–6)
*t*_1/2_ (h)	nd	nd	nd	13.8 (21%)	11.2 (25%)	9.99 (14%)
AUC_0–12h_ (ng^.^h·mL^−1^)	207 (79%)	623 (69%)	1843 (33%)	1917 (66%)	3175 (30%)	7909 (36%)
Oral clearance (L·h^−1^)	nd	nd	nd	7.24 (60%)	6.85 (33%)	5.02 (43%)
Ae_(0–12h)_ (μg)	34.6 (50%)	107 (92%)	362 (46%)	408 (46%)	644 (38%)	1345 (29%)
Ae_(0–12h)_ (%)	0.35 (50%)	0.54 (92%)	1.03 (46%)	4.08 (46%)	3.22 (38%)	3.84 (29%)

Multiple ascending dose studies were conducted in eight healthy male subjects per dose level.

a Median ± range

The concentrations of UE2343 in plasma and CSF were determined during administration of 35 mg b.i.d for 4 days. Mean C_max_ on Days 1 and 4 was reached at 5 h post‐dose (Table 5). The extent of accumulation of UE2343 in plasma after repeated daily dosing on Day 4 was 3.3‐fold, consistent with an elimination *t*_1/2_ in the order of 1 day. Between‐subject variability in the extent of systemic exposure to UE2343 was high with geometric CVs for C_max_ and AUC_0–12_ of 59 to 81%. A single CSF sample was taken from each subject at 5 h post‐dose on Day 4. The mean concentration of UE2343 in the CSF was 69.8 ng·mL^−1^, ranging from 41.2 to 99.9 ng·mL^−1^ and 7.46 to 11.9% of simultaneous total plasma levels (Table 5).

**Table 5:** Pharmacokinetic parameters in plasma and CSF following 35 mg b.i.d for 4 days

Pharmacokinetic parameter	35 mg UE2343	
	Day 1 Mean (CV%)	Day 4 Mean (CV%)
C_CSF, 5h_ (ng·mL^−1^)	nd	69.8 (44%)
C_max_ (ng·mL^−1^)	235 (60%)	708 (59%)
T_max_ (h)^a^	5.1 (4–6)	5.1 (3–5)
*t*_1/2_ (h)	6.24 (82%)	8.97 (39%)
AUC_0–12h_ (ng.h mL^−1^)	1710 (66%)	5670 (81%)

The study was conducted in four healthy subjects.

a Median ± range

### HPA axis hormones and steroids

**Figure 3:**
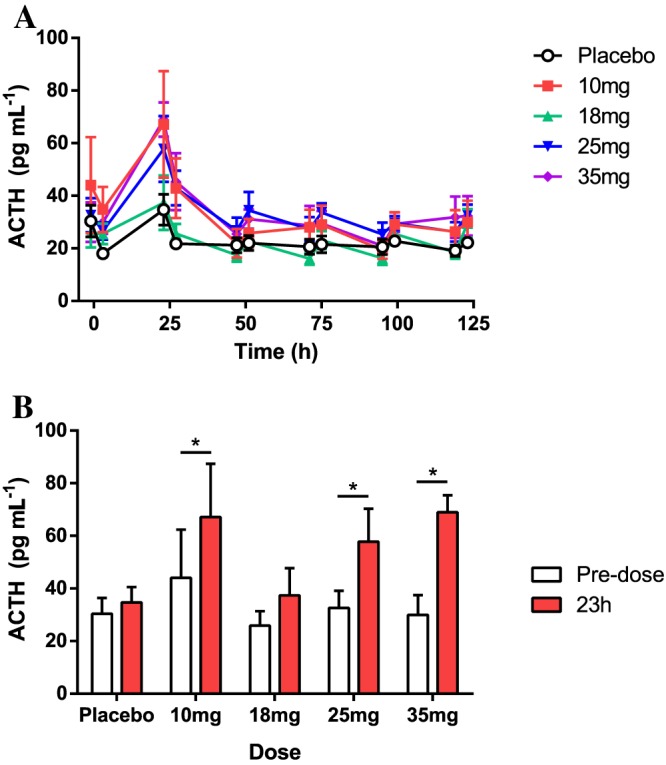
Plasma ACTH following a single oral dose of UE2343. Six healthy male and two healthy female subjects per dose level. (A) ACTH levels up to 125 h post‐dose. (B) ACTH at 23 h post‐dose; ANOVA *P < 0.05.

Following single dose administration of UE2343, there was no effect on serum cortisol, 4‐androstenedione or testosterone levels at any of the doses tested (Supporting Information Fig. S1A–C). DHEA‐s was higher following UE2343 administration when compared to placebo with statistically significant increases observed at 2 (*P* < 0.05, data not shown), 5 (*P* < 0.05, data not shown) and 35 mg (*P* < 0.05) UE2343. The increases in DHEA‐s were of similar magnitude indicating that there was no apparent dose–response relationship (Supporting Information Fig. S1D). There was a dose‐dependent increase in plasma ACTH across the 18–35 mg dose range (Figure 3A). When compared with placebo, a statistically significant increase was observed for the 35 mg dose level (*P* < 0.05). The time to maximum % change was shown to occur at a median of 23–27 h post‐dose at all dose levels except 2 mg UE2343, and values had returned to baseline by approximately 47 h post‐dose. Within‐group comparisons also revealed a statistically significant increase in ACTH 23 h post‐dose when compared with pre‐dose levels following 10 (*P* < 0.05), 25 (*P* < 0.05) and 35 mg (*P* < 0.05) UE2343, indicating stimulation of the anterior pituitary component of the HPA axis (Figure 3B).

No dose‐dependent relationship was observed for changes in cortisol, testosterone, 4‐androstenedione or DHEA‐s following multiple doses of UE2343 (Supporting Information Fig. S2A–E). For cortisol, the only statistically significant difference observed was between the 20 mg UE2343 group and placebo (*P* < 0.05) (Supporting Information Fig. S2A–B). When compared with placebo, statistically significant increases in 4‐androstenedione from baseline were observed for doses of 10 (*P* < 0.05) and 20 mg (*P* < 0.05) (Supporting Information Fig. S2C). DHEA‐s was increased from baseline, with statistically significant differences observed between the 10 mg UE2343 group and placebo (*P* < 0.05) as well as the 35 mg UE2343 group and placebo (*P* < 0.05); there was a trend towards an increase at 20 mg (*P* = 0.0505) (Supporting Information Fig. S2E). The mean maximum percentage change in ACTH (from baseline) was 222, 195 and 230% for the 10, 20 and 35 mg UE2343 doses, respectively, but these differences were not statistically significant when compared with placebo across the 13 day sampling period (Supporting Information Fig. S3).

### Pharmacodynamic inhibition of 11β‐HSD1 in the liver

**Figure 4:**
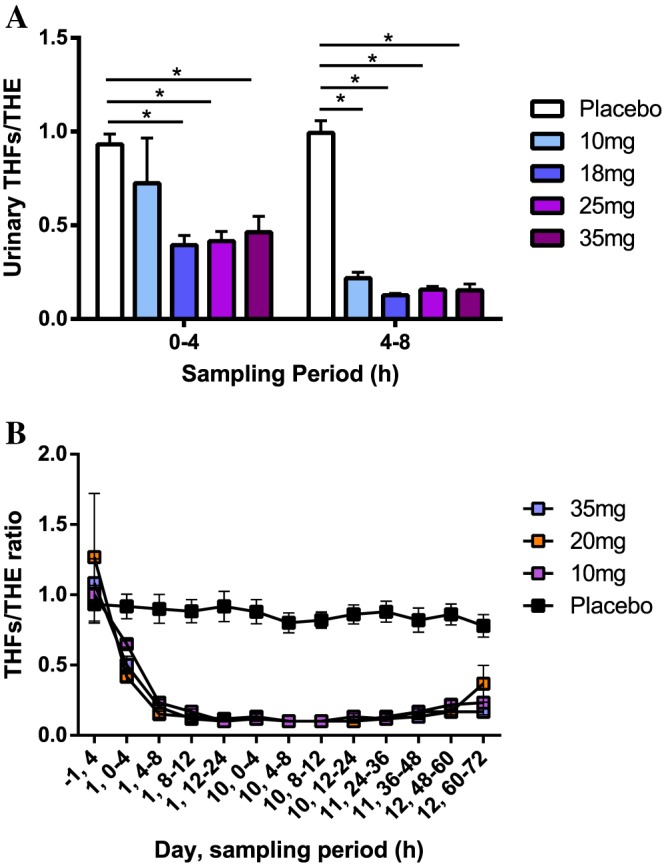
Urinary THFs/THE ratios. (A) Effects on Day 1 following a single dose of UE2343; ANOVA *P < 0.05. Six healthy male and two healthy female subjects per dose level. (B) Effects up to Day 12 following multiple doses of UE2343; ANOVA, 10 (*P* < 0.05), 20 (*P* < 0.05) and 35 mg (*P* < 0.05). Eight healthy male subjects per dose level.

11β‐HSD1 is predominantly located in the liver and adipose tissue. Cortisol (F) and cortisone (E) are cleared from the liver via the enzymes 5α‐reductase and 3α‐HSD to generate tetrahydro (TH) metabolites that are eliminated in the urine. Measurement of urinary TH metabolite ratios thus provides a surrogate measure of 11β‐HSD1 inhibition in the liver (Best and Walker, 1997). Following a single dose of UE2343, there was a statistically significant reduction in the urinary THFs/THE ratio between 0 and 4 h at doses of 18, 25 and 35 mg, equating to a reduction of 58, 55 and 50% respectively (Figure 4A). At 4–8 h, statistically significant reductions in the THFs/THE ratio of 79, 86, 83 and 83% were observed for doses of 10, 18, 25 and 35 mg respectively.

During the multiple ascending dose study statistically significant differences in the THFs/THE ratio were observed between placebo and all UE2343 treatment groups: 10 (*P* < 0.05), 20 (*P* < 0.05) and 35 mg (*P* < 0.05). Maximal reduction (84–89% reduction) in the THFs/THE ratio was achieved from 12 to 24 h on Day 1 across all dose levels and was maintained throughout the dosing period (Figure 4B).

### Estimation of 11β‐HSD1 inhibition in brain

**Figure 5:**
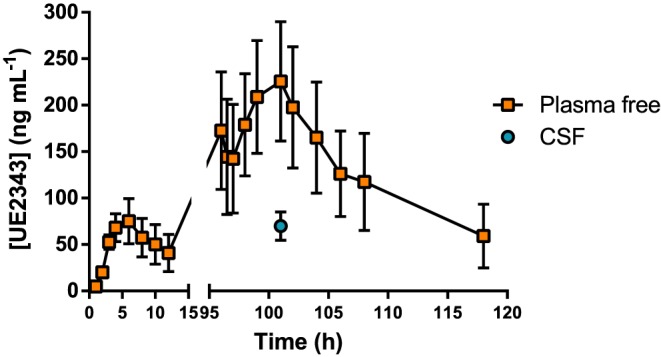
UE2343 levels in plasma and CSF following 4 days of administration of 35 mg b.i.d in four healthy male subjects.

The presence of UE2343 in the CSF samples taken from the human subjects confirmed that the compound crosses the blood brain barrier. UE2343 is 70% bound to plasma proteins; therefore, at plasma C_max_ on Day 4 (101 h), the mean UE2343 CSF levels were 33% of the free plasma levels (range: 25–40%) and 10% of the total plasma levels (range: 7–12%) (Figure 5). It was assumed that these ratios were maintained across all dose levels and time points to calculate the likely concentrations of UE2343 in the CSF at each of the dose levels used in the multiple ascending dose study. From these calculations, it was estimated that the mean CSF nadir at steady state [minimum plasma concentration (C_min_)] was 13, 22 and 50 ng·mL^−1^ at 10, 20 and 35 mg b.i.d. respectively. Maximal CSF concentrations were estimated to be 20, 34 and 81 ng·mL^−1^ at 10, 20 and 35 mg b.i.d, suggesting that steady state brain levels exceeded the IC_50_ value for UE2343 (9 ng·mL^−1^) across the dosing range.

## Discussion

A series of novel 11β‐HSD1 inhibitors were evaluated *in vitro* and *in vivo* with the objective of identifying a candidate molecule with the correct blend of characteristics for chronic oral administration in patients with AD. Physicochemical parameters for lead compounds were aligned with those required for CNS penetration. In line with CNS MPO scores, all compounds tested *in vivo* were able to access the CNS to varying degrees, with compound **8** displaying the greatest brain penetration. Notably, this compound contained a fluorine in place of a hydroxyl. Although compound **7** possessed higher *in vitro* potency towards 11β‐HSD1 and a higher brain : plasma free fraction in rats, its other properties were suboptimal compared with compound **4** (UE2343), which displayed a superior preclinical profile for further development, including lower *in vitro* clearance in human liver microsomes, lower human plasma protein binding, a greater safety margin at the human hERG channel and higher aqueous solubility. UE2343 was thus selected as a development candidate for preclinical safety assessment followed by clinical evaluation in humans.

Clinical evaluation of UE2343 in single and multiple ascending dose studies demonstrated that doses of 2 to 35 mg UE2343 once daily (q.d) and 10 to 35 mg b.i.d were safe and well tolerated in human subjects. As expected, the normal diurnal variation in plasma cortisol and overall cortisol concentrations were not altered by the administration of UE2343, with the exception of a small dose‐independent increase at 20 mg b.i.d. Regimens of 10 to 35 mg b.i.d were associated with more pronounced changes in adrenal hormone production linked to perturbation of the HPA axis, likely to be a consequence of preventing peripheral regeneration of cortisol and enhancing cortisol clearance. Following single dose administration, statistically significant changes were noted in the ACTH‐dependent adrenal androgen DHEA‐s in plasma, but no changes in the other plasma, androgens 4‐androstenedione or testosterone were observed. Curiously, at doses of 2 and 5 mg, when the level of UE2343 in plasma was below the level of quantification, statistically significant changes in the concentration of plasma DHEA‐s compared with placebo were observed, which may indicate some modulation of the HPA axis associated with 11β‐HSD1 inhibition in the periphery at low doses of UE2343. The increase in DHEA‐s was accompanied by a profound increase in ACTH at doses of 10, 25 and 35 mg q.d. indicating substantial reduction of 11β‐HSD1‐mediated cortisol regeneration in tissues.

Compensatory up‐regulation of the HPA axis in response to 11β‐HSD1 inhibition was also noted following multiple doses of 10, 20 and 35 mg UE2343. Both 4‐androstenedione and DHEA‐s were elevated, with effects persisting up to 3 days after the termination of dosing (Supporting Information Fig. S1C,E). Increases in ACTH secretion were also elevated compared with placebo, although the changes did not reach significance and no dose‐dependence was noted. The compensatory changes in ACTH secretion in response to UE2343 administration were consistent with those observed in studies of other 11β‐HSD1 inhibitors, where similar increases in the adrenal androgens 4‐androstenedione and DHEA‐s have also been noted (Rosenstock *et al.,* 2010; Feig *et al.,* 2011; Shah *et al.,* 2011; Lui *et al.,* 2013; Freude *et al.,* 2016).

Extra‐adrenal tissue generation of cortisol from cortisone by 11β‐HSD1 augments the circulating pool of cortisol, which in turn affects the net metabolic clearance of cortisol from the liver. Sampling of pooled urine over 4 h periods allowed detailed analysis of the THFs/THE ratio to determine 11β‐HSD1 inhibition in liver. Maximal inhibition (83–89% reduction in THFs/THE ratio) was achieved between 4 and 8 h following single and multiple dosing of UE2343. Substantial inhibition persisted for up to 48 h following withdrawal of UE2343 suggesting long‐lived enzyme inhibition. The results compare favourably with those reported for other 11β‐HSD1 inhibitors tested in humans; ABT‐384 was shown to decrease the THFs/THE ratio by 87–97%, BI 135585 by 65–75% and RO‐5093151 by 86–92% (Lui *et al.,* 2013; Heise *et al.,* 2014; Freude *et al.,* 2016).

UE2343 was detected in plasma at doses of 10 mg and above. Following a single dose of UE2343, the exposure increased in a greater than proportional manner between 10 and 18 mg, but the increase in UE2343 concentration between 25 and 35 mg was sub‐proportional suggesting that saturation of an absorption or distribution pathway may occur at higher doses. This effect was not noted following repeat dosing where plasma UE2343 levels were approximately dose proportional on Day 10. Renal clearance (CL_R_) was low following single or multiple dosing of UE2343, with a small increase in % Ae at day 10 to approximately 4%. The plasma T_max_ following single and multiple dosing was moderately long (3–6 h). This compares with a T_max_ = 1 h in dog and, in humans, may suggest slow dissolution of drug in the stomach or slow absorption across membranes; however, each of these phenomena would be inconsistent with the physicochemical properties of UE2343.

The ability of UE2343 to access the CNS was assessed by determining its concentration at plasma C_max_ in the CSF. Concentrations of UE2343 in the CSF ranged from 7.46 to 11.9% of total plasma levels and 25 to 40% of free plasma levels, indicating moderate penetration of UE2343 into the brain. The data were consistent with those obtained from preclinical studies in rats where the UE2343 level in brain was 43% of the free plasma concentration.

The CSF data for UE2343 compare favourably with data published for ABT‐384, for which the CSF levels were 0.3 to 1% of total plasma levels (Katz *et al.,* 2013). In this study, the authors also measured deuterated cortisol adducts in the CSF of two subjects in an effort to determine brain‐specific inhibition of 11β‐HSD1, and inferred complete inhibition of brain enzyme at levels of ABT‐384 in the CSF that were well below its cellular IC_50_ and which would not be expected to deliver significant 11β‐HSD1 inhibition. We suspect the changes in deuterated cortisol in CSF reflect inhibition of 11β‐HSD1 by ABT‐384 in liver and consequent changes in plasma deuterated cortisol enrichment. In contrast, UE2343 would be expected to achieve concentrations in CSF up to ninefold greater than its IC_50_ at C_max_ and more than fivefold its IC_50_ at C_min_ following twice daily dosing of 35 mg. Trough CSF, concentrations of UE2343 would also be expected to be higher than its IC_50_ at doses of 10 and 20 mg b.i.d. Previous preclinical studies have demonstrated that sub‐maximal inhibition of 11β‐HSD1 in the brain is sufficient to reverse memory impairments in ageing and AD (Sooy *et al.,* 2010; Sooy *et al.,* 2015). If translated to humans, it is likely that similar levels of brain 11β‐HSD1 inhibition will be sufficient to provide cognitive enhancement. The failure of ABT‐384 to achieve an improvement in cognitive function in a Phase 2 study in AD patients may reflect inadequate inhibition of brain 11β‐HSD1 (Marek *et al.,* 2014). The data described herein suggest that the requisite level of 11β‐HSD1 inhibition in brain is achievable over a prolonged period following twice‐daily oral administration of UE2343.

## Author contributions

S.P.W., J.R.S. and B.R.W. led the Wellcome Trust‐funded programme, developing and refining the experimental design. A.M., M.B. and K.S. did or designed preclinical *in vitro* and *in vivo* experiments. S.P.W., T.D.P., H.J.H. and T.R.P. did or designed the medicinal chemistry. R.A. performed urinary steroid analysis. A.B., B.R.W., V.S.R. and J.W.K. designed and reviewed clinical studies. All authors contributed to data analysis or interpretation. All authors contributed to, revised and approved the final version of the manuscript.

## Conflict of interest

This work was primarily supported by a Wellcome Trust Seeding Drug Discovery award (to B.R.W., S.P.W. and J.R.S.). S.P.W., B.R.W., J.R.S., T.D.P., H.J.H. and T.R.P. are inventors on relevant patents owned by the University of Edinburgh and licensed to Actinogen Medical Ltd. A.B. acted as medical monitor on behalf of the University of Edinburgh and Actinogen Medical Ltd. S.P.W., B.R.W., J.R.S. and A.B. act as paid consultants to Actinogen Medical Ltd.

## Declaration of transparency and scientific rigour

This Declaration acknowledges that this paper adheres to the principles for transparent reporting and scientific rigour of preclinical research recommended by funding agencies, publishers and other organisations engaged with supporting research.

## Supporting information


**Figure S1** Adrenal steroid levels following single dose administration. 6 healthy male and 2 healthy female subjects per dose level. A. Cortisol, B. 4‐androstenedione, C. testosterone and D. DHEA‐s.

**Figure S2** Adrenal steroid levels following multiple dose administration. 8 healthy male subjects per dose level. A. Day 1 cortisol, B. Day 10 cortisol, C. 4‐androstenedione, D. testosterone, E. DHEA‐s.

**Figure S3** ACTH levels following multiple dosing. 8 healthy male subjects per dose level. A. Day 1 and B. Day 10.
