# Supplementary figures and images for: Selection and early clinical evaluation of the brain‐penetrant 11β‐hydroxysteroid dehydrogenase type 1 (11β‐HSD1) inhibitor UE2343 (Xanamem™)
Source: Br J Pharmacol. 2017 Jan 25;174(5):396–408. doi: 10.1111/bph.13699 (PMC5301048; doi:10.1111/bph.13699)

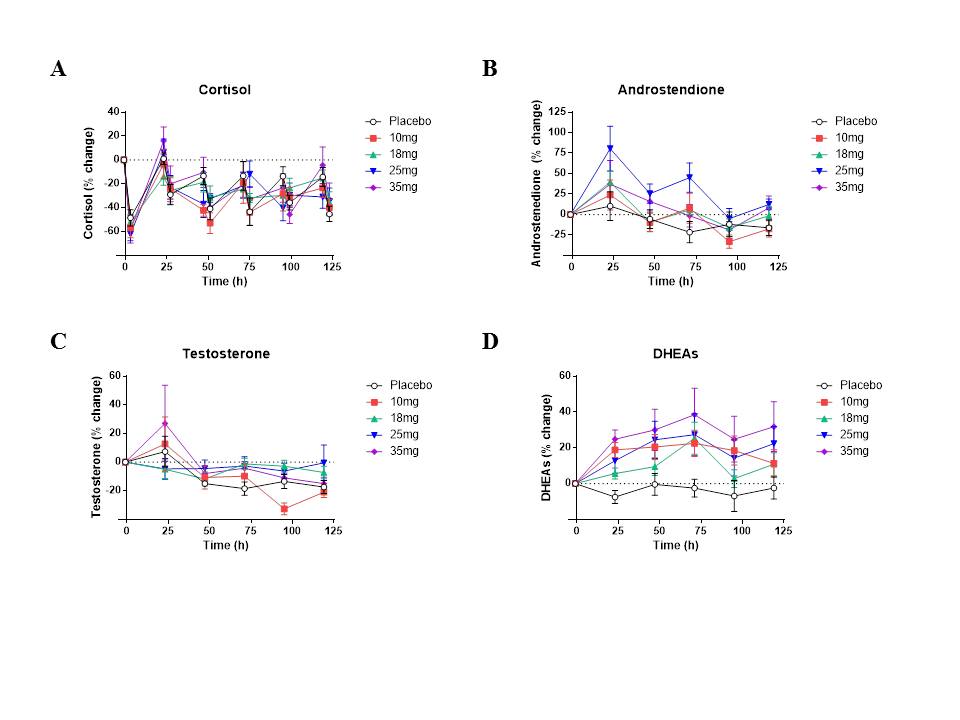

Supplement: Supplementary file 1 — Figure S1 Adrenal steroid levels following single dose administration. 6 healthy male and 2 healthy female subjects per dose level. A. Cortisol, B. 4‐androstenedione, C. testosterone and D. DHEA‐s. [file BPH-174-396-s001.TIF]

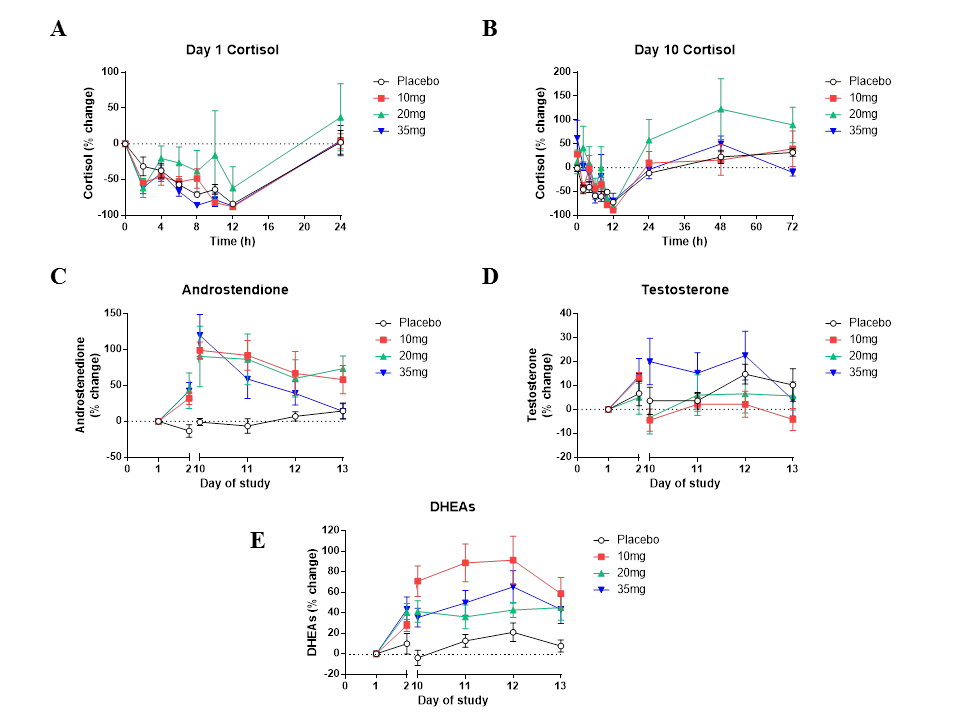

Supplement: Supplementary file 2 — Figure S2 Adrenal steroid levels following multiple dose administration. 8 healthy male subjects per dose level. A. Day 1 cortisol, B. Day 10 cortisol, C. 4‐androstenedione, D. testosterone, E. DHEA‐s. [file BPH-174-396-s002.TIF]

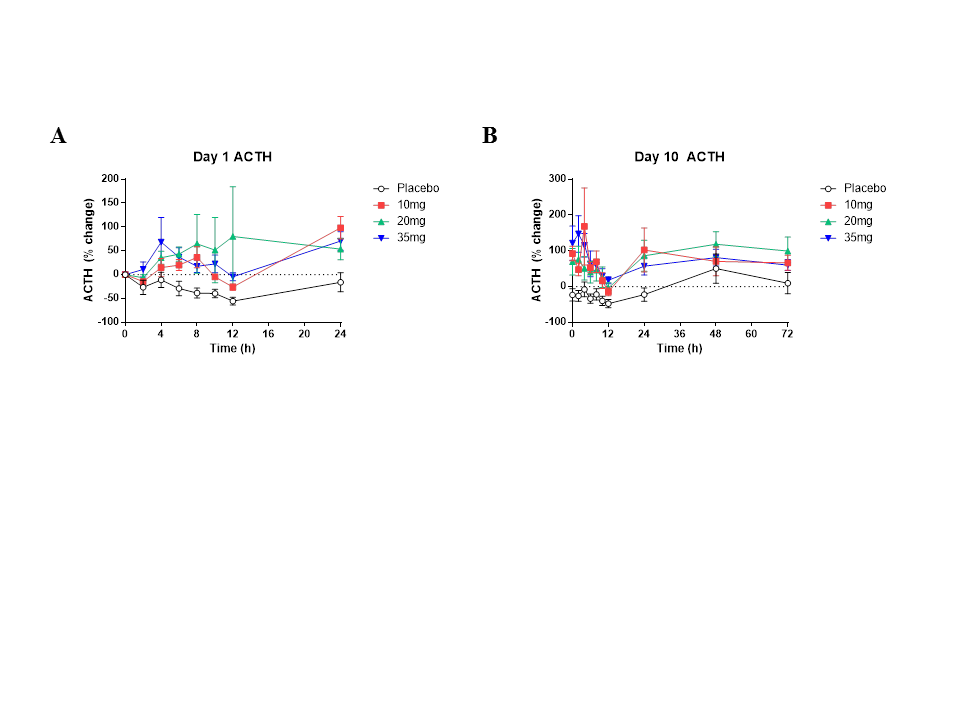

Supplement: Supplementary file 3 — Figure S3 ACTH levels following multiple dosing. 8 healthy male subjects per dose level. A. Day 1 and B. Day 10. [file BPH-174-396-s003.TIF]
